# Integrating emotional valence and semantics in the human ventral stream: a hodological account

**DOI:** 10.3389/fpsyg.2015.00032

**Published:** 2015-01-28

**Authors:** Sylvie Moritz-Gasser, Guillaume Herbet, Hugues Duffau

**Affiliations:** ^1^Department of Neurosurgery, Gui de Chauliac Hospital, Montpellier University Medical CenterMontpellier, France; ^2^Team “Plasticity of Central Nervous System, Stem Cells and Glial Tumors,” INSERM U1051, Institute for Neuroscience of Montpellier, Saint Eloi HospitalMontpellier, France

**Keywords:** ventral stream, emotion, semantic processing, anatomo-functional connectivity, brain electrostimulation mapping, subcortical pathways

## Introduction

Accessing the meaning of words to produce and understand language requires the activation of semantic representations. These latter are stored in semantic memory, organizing concepts according to semantic attributes (e.g., *the cat mews*), and semantic categories (e.g., *the cat is an animal*). Concerning semantic attributes, non-living concepts (e.g., *tools*) are processed preferentially according to functional features (e.g., *a saw cuts*) rather than visual features, whereas living concepts (e.g., *animals*) are processed preferentially according to visual features (e.g., *the cat is little, with sharp ears*) rather than functional ones (Warrington and Shallice, 1984). Within these semantic attributes, some are widely shared, independently from our personal history (e.g., *the cat mews*), and some are linked with our autobiographic memory (e.g., *I had a cat when during my childhood*). Moreover, most of the concepts have an emotional connotation which, whether it is widely shared (e.g., *a black cat brings misfortune*) or linked with our personal history (e.g., *I love cats because mine was so soft*) in a subjective-centered manner, constitutes a semantic attribute, i.e. a defining characteristic (Cato Jackson and Crosson, 2006).

Therefore, not only semantic category and “cold” widely-shared semantic attributes, but also “warm” emotion-related attributes should be activated to produce or understand a word. Even if the meaning of words may be accessed by a single “cold” semantic processing, words with emotional connotation (widely-shared and/or personal) are more quickly and efficiently processed (Bock and Klinger, 1986), allowing a faster and more accurate lexical access than neutral words (Scott et al., 2009; Méndez-Bértolo et al., 2011; Kissler and Herbert, 2013). It is worth noting that processing word-emotional connotation “differs from the actual experience of emotion: emotional connotation refers to knowledge about the emotional property of an object” (Cato Jackson and Crosson, 2006) and that “emotion modulates word production at several processing stages” (Hinojosa et al., 2010).

Semantic representations forming concepts are more than the simple summation of defining features (Lambon-Ralph et al., 2010). However, how these semantic representations are organized at the neural level is still poorly understood. While some models suggest a distributed organization between a number of interacting cortical associative regions (Turken and Dronkers, 2011), an alternative model proposes an unified organization of semantic representations in an amodal shape in the anterior temporal lobes (ATLs), receiving integrated information from different modality-specific cortical areas. In this latter framework, the ATLs are named “amodal hubs” (Patterson et al., 2007; Lambon-Ralph et al., 2009).

Here, in the light of our clinical observations during picture naming in glioma patients who underwent awake surgery, we bring a new insight on how semantic and personal-emotional information are integrated at the brain systems level, enabling to perform a well-rounded and efficient semantic processing, in order to achieve a complete noetic experience.

## A direct and an indirect route for semantic processing

We highlighted previously the crucial role of the inferior fronto-occipital fasciculus (IFOF) in semantic processing (Duffau et al., 2013; Moritz-Gasser et al., 2013; Almairac et al., 2014). We proposed this long-association pathway, which comes from the occipital lobe, posterior-lateral temporal areas and parietal cortex, and runs to the orbitofrontal and dorsolateral prefrontal cortices (Catani et al., 2002; Kier et al., 2004; Wakana et al., 2004), as a ventral plurimodal direct route for semantic processing, parallel to an indirect route subserved by the complex inferior longitudinal/uncinate fasciculi (ILF/UF). Indeed, intraoperative mapping during awake surgery for brain glioma (Duffau et al., 2002, 2005) shows that direct electrostimulation of the left IFOF during a naming task always induces semantic disorders (semantic paraphasias or anomias). This semantic disorganization may be either plurimodal (verbal and non-verbal) when stimulating the deep layer of the IFOF, evidenced by the inability for the patient to perform a non-verbal semantic association task, or “only” verbal, when stimulating the superficial layer of the IFOF. Recent studies, based on the Klingler fiber dissection technique, identified two different components of the IFOF: a superficial and dorsal subcomponent, which connects the dorsolateral prefrontal lobe with the superior parietal lobe and the posterior portion of the superior and middle occipital gyri; and a deep and ventral subcomponent, which connects the orbitofrontal cortex with the posterior portion of the inferior occipital gyrus and the posterior temporal-basal area (Martino et al., 2010; Sarubbo et al., 2013). This multilayer organization of the IFOF has recently been confirmed by q-ball tractography (Caverzasi et al., 2014). Interestingly, these anatomical descriptions correspond with the cortical network involved in semantic control, namely pre-frontal, temporal-basal and parietal areas (Whitney et al., 2011).

Thus, we assumed that the IFOF plays a crucial role in the monitoring of multimodal semantic processing, and we proposed a dynamic dual-stream model of the ventral amodal semantic route, including both the deep and the superficial layers of the IFOF and the indirect (ILF/UF) ventral pathway (Duffau et al., 2013). Based on data issued from intraoperative electrostimulation, we suggested that the IFOF might play a crucial role not only in multimodal semantic processing but beyond, in the awareness of conceptual knowledge, namely noetic consciousness (Moritz-Gasser et al., 2013).

Tractographic studies suggested that semantic processing is underlain by the sole complex ILF/UF (Agosta et al., 2010). The ILF has a vertical component in the parietal lobe, and a horizontal component that lies within the white matter of the occipital and inferior temporal regions (Schmahmann et al., 2007). From the dorso-lateral surface of the occipital lobe, the ILF runs ventro-medially from the posterior lingual and fusiform gyri and dorso-medially from the cuneus. Then the branches run forward to the superior, middle and inferior anterior temporal gyri on the lateral surface, and medially to the amygdala and the parahippocampal gyrus (Catani et al., 2003; Martino and de Lucas, 2014). The ILF seems to be implicated in visual perception, face and object recognition (Catani and Mesulam, 2008a; Fox et al., 2008), reading (Epelbaum et al., 2008) and spoken language (Mummery et al., 1999; Catani and Mesulam, 2008b).

Concerning face/object recognition and reading, it seems that only the posterior part of the ILF (“visual part” corresponding to occipito-inferotemporal fibers) is involved, whereas concerning spoken language (naming), both posterior and anterior parts of the ILF are involved (the former in visual processing of the object or picture, and the latter in “linking object representations to their lexical labels” (Catani and Mesulam, 2008b), by “allowing the semantic system access to stored lexical information” (Foundas et al., 1998; Mummery et al., 1999).

The UF is a ventral associative bundle that connects the ATL and amygdala with the orbitofrontal cortex (Catani et al., 2002; Catani and Thiebaut de Schotten, 2008). It runs inferiorly to the IFOF within the temporal stem, then it splits into a large ventro-lateral branch which terminates in the lateral orbitofrontal cortex and a smaller medial branch which terminates in the frontal pole (Catani et al., 2002; Thiebaut de Schotten et al., 2012). The UF is traditionally considered to be part of the limbic system (Catani et al., 2013; Von Der Heide et al., 2013). Given its connections, functions linked to the UF may concern episodic memory (value-based updating of stored representations), language (retrieval of proper names for people, some aspects of semantic memory retrieval), and social-emotional processing (valuation of stimuli, emotional meaning of concepts) (Von Der Heide et al., 2013).

We postulate that this indirect pathway (ILF/UF) is involved but not sufficient to perform an efficient semantic processing. We propose that, given their respective cortical terminations, one of the roles of the complex ILF/UF might be to convey critical emotional and mnemonic information associated with words and needed to generate well-rounded supramodal representations of concepts, under the amodal control of the IFOF.

## Cortical network and subcortical connectivity of personal emotional-valued semantic processing during lexical access: proposal of a hodotopical model

Picture naming requires an early visual processing and recognition by accessing a stored structural description, and then the selection of the corresponding semantic representation or “concept.” In parallel with this preverbal processing, appropriate lexical representations or “words” are activated (Ferrand, 1997; Levelt, 2001), thanks to the selection of the most accurate defining features of the semantic representation (Papagno, 2011). Within these defining features or “semantic attributes,” some are “cold,” widely-shared, and some are “warm,” i.e., with an emotional value, itself widely-shared or personal. As mentioned, words with emotional connotation are processed faster and more efficiently than neutral words.

We hypothesize that, if we can access words accurately with only “cold” attributes processing, a well-rounded lexical access will be achieved more efficiently thanks to an integrated processing of words-related emotion. We argue that the indirect ventral semantic stream, subserved by the complex ILF/UF, is the anatomical substrate of this high-level processing, while the direct ventral semantic stream, subserved by the IFOF, is crucial in the monitoring of amodal semantic processing. Thus, we propose an original anatomo-functional model of lexical access, in which all processes (except the early visual processing) are performed in parallel and synchronically.

**Figure 1:**
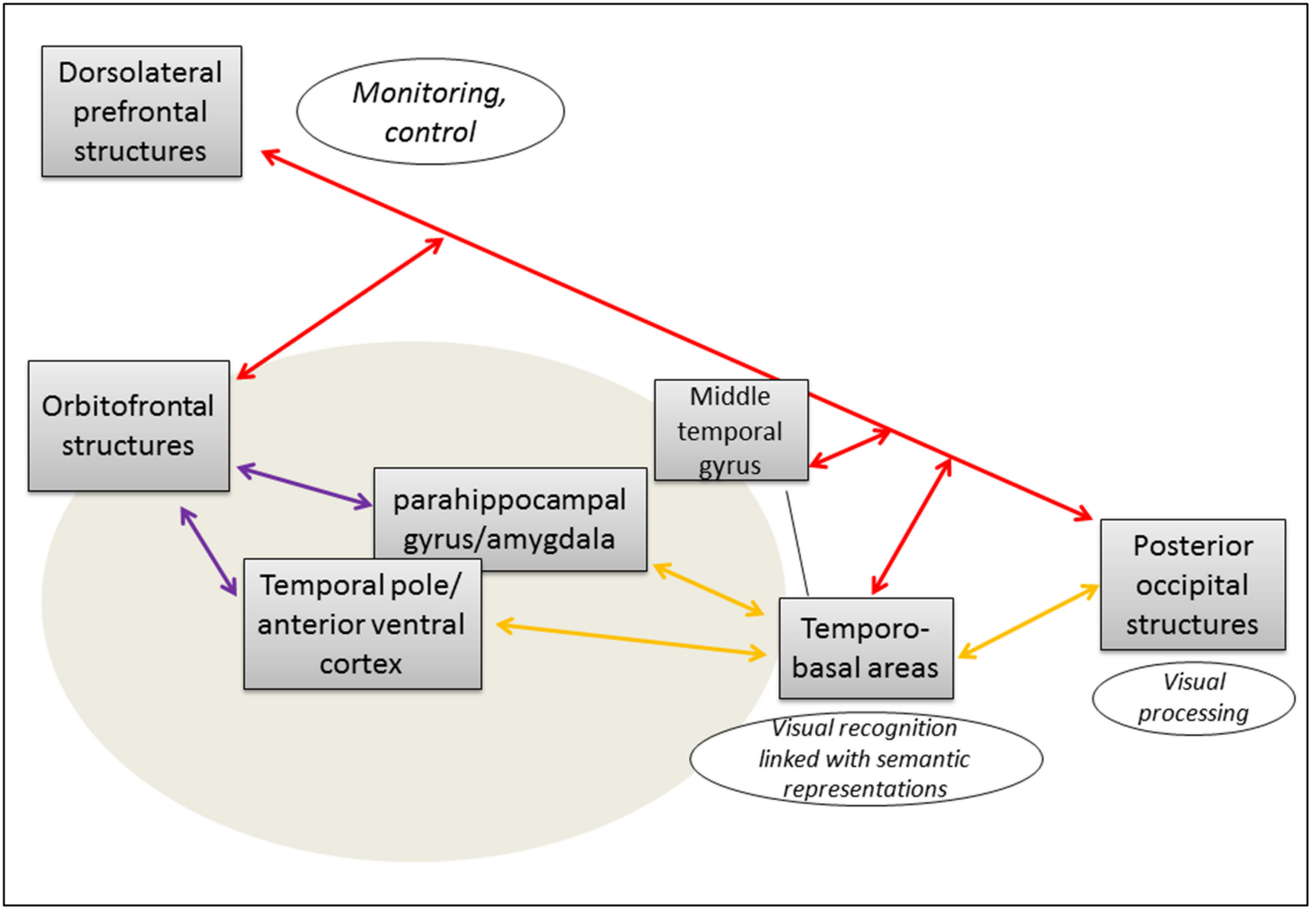
**Cortical network and subcortical connectivity of emotional-valued semantic processing during lexical access**. Proposal of an anatomo-functional model (the gray oval signalize the network specifically involved in lexical access of emotionally connoted words). 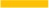 Inferior longitudinal fasciculus (ILF); 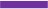 uncinate fasciculus (UF); 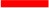 inferior fronto-occipital fasciculus (IFOF). Integration of processes from both the indirect (ILF/UF) and the direct (IFOF) ventral streams is required for an efficient emotional-valued word semantic processing.

Visual processing in occipital structures leads to visual recognition thanks to the activation of structural descriptions stored in temporo-basal areas, linked with corresponding semantic representations. During this preverbal stage, information is transmitted via the posterior part of the ILF. Then, to select the appropriate word, corresponding lexical representations are activated following “cold” and “warm” defining features of the semantic representation thanks to a synchronous processing involving the middle temporal gyrus, anterior ventral temporal cortex and temporal pole via the anterior part of the ILF—interacting with orbitofrontal structures via the UF. These parallel processes are supervised and controlled via the IFOF, in an amodal way (Figure 1).

Interestingly, the left ATL seems to be involved in the retrieval of people proper names (Damasio et al., 1996; Papagno and Capitani, 1998; Grabowski et al., 2001). Our model may explain some clinical presentations to the extent that people proper names can only be accessed with their emotional connotation.

Furthermore, it is worth noting that some parts of the distributed cortical network our model highlights have previously been proposed as being involved in the processing of word emotional valence in an fMRI study (Kuchinke et al., 2005).

Finally, one of our previous studies based on intraoperative electrostimulation (Mandonnet et al., 2007) suggested that the ILF was not essential in language processing. We proposed that “due to plasticity phenomena induced by slow growing lesion, the function could have been redistributed over the ipsi- or contralateral hemisphere.” In other words, the complex ILF/UF is possibly not crucial in semantic processing (because, as mentioned above, an acceptable semantic processing may be performed following only “cold” semantic attributes), and compensable in brain lesions, but this complex is necessary in normal conditions to perform well-rounded, fast and efficient emotional-valued lexico-semantic processing. Nonetheless, the IFOF remains in our model the critical substrate subserving the monitoring and the control of amodal semantic processing. This repeated assumption is in line with the hypothesis of a semantic working memory pathway via the IFOF (Turken and Dronkers, 2011).

In summary, only the integration of synchronous processes from both the indirect and direct ventral streams allows an accurate, efficient and emotionally connoted semantic processing.

## Conclusion

We propose an original anatomo-functional model of lexical access, integrating the processing of personal emotional values of words. This model, based on clinical observations of glioma patients undergoing awake surgery and on an extensive review of the literature concerning the anatomo-functional descriptions of white matter associative tracts, puts forward the implication of a large-scale distributed network in this processing. This network might consist of the indirect semantic ventral stream, namely the complex ILF/UF, interconnecting infero-temporo-occipital areas and antero-ventral and medial temporal areas with orbitofrontal structures, which would act synchronically under the amodal monitoring of the direct ventral stream underlain by the IFOF. Integration of processes from both the indirect and direct ventral streams would be required to achieve an emotion-tinged semantic processing, fully and solely human. We may assume that a sole “cold” semantic processing, devoid of any emotional connotation, would entail a disembodied communication, not allowing making sense to situations and to the whole world around us. In other words, a sole “cold” semantic processing wouldn't be a human semantic processing, rich, complex and linked with personal history. We then propose that integration of processes from both the indirect and the direct ventral streams allows a fully achieved, human semantic processing leading to a complete noetic experience.

### Conflict of interest statement

The authors declare that the research was conducted in the absence of any commercial or financial relationships that could be construed as a potential conflict of interest.
